# Educational Attainment and Staphylococcus aureus Colonization in a Hispanic Border Community: Testing Fundamental Cause Theory

**DOI:** 10.1128/mSphere.00623-20

**Published:** 2020-09-30

**Authors:** Steven D. Barger, Monica R. Lininger, Robert T. Trotter, Heidi A. Wayment, Mimi Mbegbu, Shari Kyman, Talima Pearson

**Affiliations:** a Department of Psychological Sciences, Northern Arizona University, Flagstaff, Arizona, USA; b Department of Physical Therapy and Athletic Training, Northern Arizona University, Flagstaff, Arizona, USA; c Center for Health Equity Research, Northern Arizona University, Flagstaff, Arizona, USA; d Department of Anthropology, Northern Arizona University, Flagstaff, Arizona, USA; e Pathogen & Microbiome Institute, Northern Arizona University, Flagstaff, Arizona, USA; University of Nebraska Medical Center

**Keywords:** socioeconomic status, *Staphylococcus aureus*, health status disparities, Southwestern U.S., Hispanic Americans

## Abstract

This study was carried out to evaluate hypotheses generated by fundamental cause theory regarding the socioeconomic status (SES) gradient in colonization with Staphylococcus aureus among Hispanic and non-Hispanic adults living in a border community. Participants (*n* = 613) recruited in naturally occurring small groups at public and private sites throughout Yuma County, AZ, completed a sociodemographic survey and swabbed their palms, noses, and throats to sample microbial flora. Positive S. aureus colonization among non-Hispanic white participants was nominally higher (39.0%; 95% confidence interval [CI] = 32.4 to 46.1%) than that in Hispanics (31.3%; 95% CI = 26.4 to 36.8%), but there was no education gradient for the sample overall (incidence rate ratio = 1.00; 95% CI = 0.90 to 1.12) or within each ethnic group separately. The education gradient between Hispanic and non-Hispanic whites was statistically equivalent. Results were consistent when home ownership was used as the SES indicator. These data show that S. aureus colonization is not linked to two different SES indicators or Hispanic ethnicity. S. aureus colonization may be considered a less preventable health risk that is outside the influence of SES-based resources.

**IMPORTANCE** Unlike some types of S. aureus infections, S. aureus colonization is not associated with ethnicity or educational attainment and thus may be outside the influence of socioeconomic status-based resources typically mobilized to avoid or mitigate preventable health risks. This assessment of a clinically silent risk that usually precedes infections may illustrate a boundary of fundamental cause theory.

## Abstract (precis)

Unlike some types of S. aureus infections, S. aureus colonization is not associated with ethnicity or educational attainment and thus may be outside the influence of socioeconomic status-based resources typically mobilized to avoid or mitigate preventable health risks. This assessment of a clinically silent risk that usually precedes infections may illustrate a boundary of fundamental cause theory.

## INTRODUCTION

Socioeconomic resources such as education and income, as well as ethnicity and race, are strongly associated with morbidity and mortality (1–4). Although health risk factors change over time, the health advantage conferred by socioeconomic resources persists. For this reason, socioeconomic resources are theorized to be “fundamental causes” of health that affect multiple disease mechanisms (5–8). Fundamental causes reflect access to resources that may be used to avoid health risks or ameliorate the consequences of health problems once they occur (5). Much of the literature on fundamental cause examines mortality risk and reflects mainly chronic diseases. Less is known about how fundamental causes relate to infectious disease (9), which accounts for substantial morbidity and mortality in the United States (10–12). One infectious agent, Staphylococcus aureus, is a bacterium that lives in close association with humans both as a harmless member of our microbial community and as a pathogen. Almost one-third of healthy Americans are colonized with S. aureus (13). This carriage almost always precedes development of infection that occurs when these bacteria penetrate outer skin layers or mucosa (14). As a pathogen, S. aureus can infect local tissues, spread to distant organs, and cause more serious systemic infections (15, 16), which, in 2017, were responsible for over 19,000 U.S. deaths (17). Importantly, over 80% of S. aureus isolates from clinically infected persons are genetically identical to strains found in those individuals’ nares (18, 19), the primary ecological niche for S. aureus in humans. Thus, colonization with S. aureus provides an important intermediary to evaluate health risk.

Infections due to S. aureus have been associated with lower socioeconomic status (SES) indicators (20–22); however, whether this extends to S. aureus colonization has not been previously addressed. Examining the distribution and determinants of S. aureus colonization is especially interesting, as colonization, in itself, is not detrimental to health but may provide important insights into developing novel prevention strategies for S. aureus infection. In addition, the clinical silence of S. aureus colonization, coupled with the strong link to subsequent infections, represents a useful context to examine predictions and boundaries of fundamental cause theory. Toward this end, we examined the association of a key socioeconomic status (SES) indicator, educational achievement, with asymptomatic S. aureus colonization in a predominantly Hispanic border community in Arizona. Yuma County, in southwestern Arizona, has a population of 213,787 (2019 estimate) (23), with Hispanics constituting approximately 64% of the population and non-Hispanic whites 31%. This region is medically underserved and as of March 2020 has the second highest unemployment rate (14.8%) of 389 metropolitan areas in the country (24).

The primary goal of the present study was to first evaluate whether the robust educational gradient in health extends to S. aureus colonization. Evidence for this gradient would be consistent with a large body of literature showing a health advantage for higher educational attainment (2, 7, 25), including S. aureus infections (20–22). In contrast, the absence of a gradient would indicate that S. aureus colonization is not subject to socioeconomic stratification and may thus require different strategies for control. Second, our goal is to examine whether the presence of other fundamental social determinants of health, such as ethnic minority status, modifies the salutary association of education. That is, social characteristics such as race and ethnicity are hypothesized to weaken the SES gradient in health (26). Thus, a more refined prediction of fundamental cause theory is that the education gradient should be present for non-Hispanic whites but absent or weaker among Hispanics. To test our research questions, we evaluated the education gradient for the sample overall and for Hispanic and non-Hispanic white groups separately. These comparisons permitted evaluation of several working hypotheses (27). If education is related to colonization overall (hypothesis 1), this finding would generally support fundamental cause theory. In contrast, a larger education gradient among non-Hispanic whites than Hispanics (hypothesis 2) would offer support for the more refined fundamental cause prediction—that education-based SES advantages are limited by Hispanic ethnicity. The absence of an education gradient for the full sample and for both groups separately would disconfirm both the general and more refined fundamental cause predictions and indicate S. aureus colonization is not sensitive to SES as indicated by educational attainment. The latter pattern would support the conclusion that S. aureus colonization, but not infection, represents a boundary of SES influence. As a sensitivity analysis, we repeated these hypothesis tests using home ownership as the SES indicator. Home ownership is a marker of wealth and is associated with longevity, independent of education and income (28, 29). Home ownership thus complements the focal analysis using education as the SES indicator.

## RESULTS

Descriptive characteristics of the sample are presented in Table 1. Overall, 34.4% (95% confidence interval [CI] = 30.8 to 38.3%) of participants were colonized with S. aureus. Colonization among non-Hispanic participants was nominally higher (39.0%; 95% CI = 32.4 to 46.1%) than that among Hispanics [31.3%; 95% CI = 26.4 to 36.8%; difference = 7.7%; 95% CI = −0.01 to 0.16; F(1, 231) = 3.36, *P = *0.068]. Education was not associated with colonization in the full sample (incidence rate ratio [IRR] = 1.00; 95% CI = 0.90 to 1.12), and this pattern persisted after adjustment for age, sex, and ethnicity (Table 2). Education was not statistically associated with S. aureus colonization when analyses were restricted to Hispanics or non-Hispanic whites with (Table 2) or without adjustment for age and sex (data not shown). Thus, tests of the fundamental cause (hypothesis 1) and of the refined fundamental cause prediction that a smaller education coefficient would be observed for minority versus majority ethnicity (hypothesis 2) were not confirmed. The education coefficients across the two groups (Table 2) were not statistically different. [χ^2^(1) = 1.79; *P = *0.181]. Male sex was the only variable consistently associated with S. aureus colonization.

**TABLE 1:** Sample baseline characteristics

Characteristic	Total	S. aureus negative	S. aureus positive
Participants, n	613	402	211
Age (yr) [mean (SD)]	32.1 (13.2)	32.6 (13.4)	31.2 (12.8)
Female sex, *n* (%)	346 (56.4)	241 (60.0)	105 (49.8)
Race/ethnicity, *n* (%)^*a*^			
Hispanic	367 (59.9)	252 (62.7)	115 (54.5)
Non-Hispanic white	246 (40.1)	150 (37.3)	96 (45.5)
Marital status [*n* (%)]			
Married/cohabiting	299 (48.8)	195 (48.5)	104 (49.3)
Divorced/separated	44 (7.2)	34 (8.5)	10 (4.7)
Widowed	10 (1.6)	9 (2.2)	1 (0.5)
Never married	258 (42.1)	163 (40.5)	95 (45.0)
Missing	2 (0.3)	1 (0.02)	1 (0.5)
Education level [*n* (%)]			
<High school	173 (28.2)	114 (28.4)	59 (28.0)
High school diploma	82 (13.4)	53 (13.2)	29 (13.7)
Some college	239 (39.0)	157 (39.1)	82 (38.9)
College graduate or higher	119 (19.4)	78 (19.4)	41 (19.4)
Home ownership [*n* (%)]			
No	229 (37.4)	156 (38.8)	73 (34.6)
Yes	371 (60.5)	240 (59.7)	131 (62.1)
Missing	13 (2.1)	6 (1.5)	7 (3.3)
Employed [*n* (%)]	440 (71.8)	286 (71.1)	154 (73.0)

a Hispanic and non-Hispanic participants were included if for race they chose “white” or “no preferred race.”

**TABLE 2:** IRR and CI for S. aureus colonization by educational attainment^*a*^

Characteristic	Overall (*n *= 613)		Hispanic (*n *= 367)		Non-Hispanic white (*n *= 246)	
	IRR	95% CI	IRR	95% CI	IRR	95% CI
Education	0.99	0.88–1.10	1.02	0.88–1.18	0.89	0.77–1.03
Age	0.99	0.98–1.00	0.98	0.97–1.00	1.01	1.00–1.02
Male sex	1.27	1.01–1.59	1.32	0.97–1.81	1.15	0.84–1.59
Non-Hispanic white	1.22	0.96–1.55				

a Education coefficients are not statistically different across Hispanic and non-Hispanic white groups [χ^2^(1) = 1.79; *P *= 0.181].

In follow-up analyses, colonization with S. aureus was not statistically associated with home ownership. Those who owned their own home had colonization prevalence (35.3%; 95% CI = 29.9% to 41.2%) similar to that of those who did not (31.9%; 95% CI = 258% to 38.7%). This association persisted in multivariate analyses that included age, sex, and ethnicity (Table 3). The home ownership regression coefficients for Hispanics and non-Hispanic whites were not statistically different [χ^2^(1) = 0.47; *P = *0.493] (Table 3).

**TABLE 3:** IRR and CI for S. aureus colonization by home ownership^*a*^

Characteristic	Overall (*n *= 600)		Hispanic (*n *= 361)		Non-Hispanic white (*n *= 239)	
	IRR	95% CI	IRR	95% CI	IRR	95% CI
Home ownership	1.13	0.88–1.46	1.17	0.85–1.60	0.99	0.67–1.45
Age	0.99	0.98–1.00	0.98	0.97–0.99	1.01	0.99–1.02
Male sex	1.30	1.03–1.65	1.33	0.97–1.83	1.24	0.88–1.73
Non-Hispanic white	1.22	0.96–1.54				

a Home ownership coefficients are not statistically different across Hispanic and non-Hispanic white groups [χ^2^(1) = 0.47; *P* = 0.493].

## DISCUSSION

We evaluated whether the robust SES gradient found in numerous types of health risk extends to colonization with a common bacterium, S. aureus, in a community sample on the United States–Mexico border. Using predictions derived from fundamental cause theory, we found no evidence for an education gradient in S. aureus colonization in the overall sample or for Hispanics and non-Hispanic whites considered separately. The refined fundamental cause model predicted that the education association would be weaker for non-Hispanic whites than Hispanics (hypothesis 2). A direct comparison of these coefficients did not confirm this prediction, a pattern also replicated using home ownership as another marker of SES.

S. aureus is a prevalent cause of clinical infection in a variety of selected populations (14), with infection almost always being traceable to identical S. aureus colonization genotypes (18, 19). While infection has been linked to SES (20–22), this study provides preliminary evidence that colonization is not linked to two different SES indicators (educational attainment and home ownership) or Hispanic ethnicity. Longitudinal studies have demonstrated that about 20% of healthy individuals are persistently colonized with S. aureus, about 60% are intermittently colonized, and about 20% are almost never colonized (30). Persistently colonized individuals carry a greater pathogen load and are at greater risk of infection, because they are continuously shedding large amounts of S. aureus (14). In this study, our cross-sectional sampling likely detected most persistent and some intermittent carriers. Our sampling at three different body sites likely increased our ability to detect intermittent carriers. While persistent carriage may be more epidemiologically important, we could not stratify by carrier status, and this is a limitation of the study.

Our results suggest that colonization in general may be categorized as a less preventable health risk because it lies outside the influence of SES-based resources that can otherwise be mobilized to avoid preventable health risks or reduce the sequelae of health problems once they start. The pattern of results found in this study contrasts with SES gradients observed for antibiotic-resistant S. aureus infections, S. aureus bacteremia (20–22), and the chronic diseases examined in previous tests of fundamental cause theory (8, 26, 31). Although modest educational differences in S. aureus colonization have been observed in previously published studies, the associations were not linear and were not observed for income (32). Nonlinear associations have also been observed for S. aureus colonization among children, but only for antibiotic-resistant S. aureus and not overall colonization (33). To our knowledge, ours is the first evaluation of the more refined fundamental cause prediction (hypothesis 2) in the context of colonization with an infectious agent.

Given evidence that S. aureus infection, but not colonization, is stratified by SES, further evaluation of SES gradients for clinical events is warranted. These include less severe and more common skin and soft tissue infections (SSTI), infections caused by antibiotic-sensitive versus -resistant S. aureus strains, and both new and recurrent S. aureus infections. To the extent that antibiotic-resistant infections and recurrent infections represent a more preventable health risk, they should be more sensitive to SES stratification.

Our study examined two SES indicators, and while our data suggest that educational attainment and home ownership are not associated with colonization, other SES markers may be. Education was the focal SES indicator in other major studies of fundamental cause theory (26, 31), including a study of S. aureus bacteremia (21). Home ownership is less often evaluated and captures resources that are distinct from education and income (28, 29). We did not assess income, but available evidence reveals no income gradient in colonization (32). Most studies examining minority modification of SES have contrasted black and white racial groups, and thus this study contributes by examining Hispanic ethnicity and expanding the inquiry to infectious disease agents. Our study setting, a border area of southern Arizona, provides a novel context to investigate carriage, dissemination, and transmission of infectious diseases.

Our study is subject to a number of limitations and caveats. Other markers of socioeconomic status may be more strongly associated with colonization than our two SES indicators. The comparison of Hispanic and non-Hispanic ethnicity is less common in the fundamental cause literature, and it is important to note the relatively young age of our sample. Our test of fundamental cause theory was situated in a community with a higher percentage of persons with Hispanic ethnicity compared to non-Hispanics, a context which could modify the SES resources with health risk (26).

Previous research shows an SES gradient for some types of S. aureus infections, and this association suggests distinct strategies for prevention, i.e., infection control methods like limiting sharing and following hygiene recommendations. In contrast, our study of colonization (rather than infection) does not show an SES gradient and indicates that colonization may not be an outcome stratified by SES. Future research should further explore the possibility of an SES link specifically for persistent carriers and whether social interactions, social contact, cultural differences in social structure, frequency of contact with colonized individuals, and general hygiene practices can impact colonization. Further exploration is also needed to examine S. aureus infection and colonization in relation to SES and minority group membership to further clarify the boundaries of SES influence and delineate strategies for infection control.

## MATERIALS AND METHODS

### Recruitment and participants.

We report data on 613 adult (≥18 years old) participants who were recruited in Yuma County, Arizona, during 2018 and 2019. The analytic sample included persons who identified as either non-Hispanic white or Hispanic white (and not Black or another race). Participants were recruited in naturally occurring small groups at public and private sites throughout Yuma County based on a theoretical sampling frame (34, 35). Examples of “public” recruitment sites include public parks with recreational facilities for individuals and families, shopping malls with outdoor public spaces, and strip malls with spaces outside major box stores. “Private space” examples include retail business spaces, individual offices in schools and nongovernmental organizations, and individual homes, with appropriate recruitment and consenting processes. This targeted sampling approach allowed us to capture a variety of relationships and mixtures of relationships that include acquaintances, friends, coworkers, and family, with various levels of contact and social intensity. Informed consent was obtained from all participants, and the Institutional Review Board of Northern Arizona University approved this project (number 1116783).

Data were collected over a 2-year period. In the first year, data were collected on electronic tablets, whereas in the second year, data were collected on paper surveys. This study is part of a larger effort to understand S. aureus colonization and transmission in social groups (36). To maintain anonymity, participants were given letters to identify themselves and other group members. As a data quality control measure, we retained for analysis only persons in groups where all group members consistently identified relationships among the group members: e.g., a person had to correctly identify themselves; persons identified as a sibling had to reciprocate that identification; their reported group size had to match the size recorded by study staff, etc. This left 633 (of 1,269) participants from the tablet-based data collection (36) and 340 (of 354) from the paper-based data collection. Of these 973 participants, we restricted our analysis to those 18 and over (*n *= 839) who had completed a screening for S. aureus (*n *= 824). We further restricted our groups to those reporting white race (*n *= 459) or “no preferred race” (*n *= 154) and who also responded to the question regarding Hispanic ethnicity (yes/no) (*n *= 613 in 232 unique groups).

Participants reported sociodemographic characteristics, including their education level and whether they owned their own home (“Regarding your primary residence, does your family own your home, rent your home, or have some other arrangement?”). Persons renting, having some other arrangement, or not knowing were classified as not owning their home. We used home ownership as an alternative SES indicator in sensitivity analyses. Income was not assessed.

### Biological-sample collection.

After recruitment and consent, participants were given a sampling kit containing BBL culture swabs and guided through swab handling and swabbing methods. Participants swabbed one of their palms, both anterior nares (with the same swab), and their throats. Each body site was swabbed for approximately 20 s. Swabs were immediately placed on ice or stored in a refrigerator at ∼4°C. Within approximately 48 h, each swab was streaked on one CHROMagar S. aureus plate, which was incubated at 37°C and read after 24 to 26 h. The medium in these plates is specific to Gram-positive bacteria but contains chromogens that turn S. aureus colonies fuchsia for easy, accurate, and highly sensitive identification (37). Additional details of the collection and processing of biological samples were reported previously (36). Any individual with a positive S. aureus culture from a swab collected from any of the three body sites was recorded as being colonized with S. aureus.

### Data analysis.

We first evaluated the association of education with S. aureus colonization in the full sample (hypothesis 1). We then evaluated the association of education with S. aureus colonization within each ethnic group (non-Hispanic white and Hispanic) separately. To provide the clearest ethnic group comparison, we cross-classified persons as non-Hispanic white (*n *= 246) if they described themselves as not Hispanic and either white (*n *= 234) or “no preferred race” (*n *= 12). We classified persons as Hispanic (*n *= 367) if they described themselves as Hispanic and either white (*n *= 225) or “no preferred race” (*n *= 142). The small number of persons describing themselves as black/African-American, Asian, Native American, Hawaiian/Pacific Islander, other race or multiracial was insufficient for separate analysis and were excluded. To evaluate whether Hispanic ethnicity attenuates the education gradient, we directly compared the education coefficients from these two models using seemingly unrelated estimation (38). This is the focal test for hypothesis 2, which examines whether the education gradient for colonization differs across ethnicity. Because participants were recruited in small, naturally occurring social groups, with the possibility of colonization status intercorrelation, all variance estimates incorporate clustering at the group level. The intraclass correlation of staphylococcus results within the groups was 0.11 (95% CI, 0.02 to 0.21).

We evaluated and confirmed the interval assumption for the single education variable. This indicates that education can be modeled as a single variable with four categories (less than high school, high school graduate, some college, and college graduate or higher). We conducted a variety of sensitivity analyses, which included covarying ethnicity (full sample only), age, and sex. We also evaluated the education gradient using a variable that combined the “less than high school” and “high school graduate” categories. Results were robust across these models. We repeated these analyses substituting a binary home ownership variable as the SES indicator.

We estimated the education gradient for S. aureus colonization using a generalized linear model with a Poisson distribution, a log link function, and robust clustered variance estimates (39). This approach produces incidence rate ratios (IRR), which are preferable to odds ratios generated from logistic regression, in part because of scaling issues for odds ratios (40) and because IRRs represent a more easily interpreted measure of association; the ratio of the proportion of S. aureus colonization in the exposed group over the proportion in the unexposed group. Odds ratios would also overestimate the association with the relatively large (>30%) prevalence of S. aureus colonization (41). Ethnicity was coded “yes” or “no,” reflecting non-Hispanic white or Hispanic participants. We used Stata 16.1 (Stata Corp., College Station, TX) for all analyses. Differences were considered statistically significant if the two-tailed *P* value was ≤0.05.
